# Exfoliation of titanium nitride using a non-thermal plasma process

**DOI:** 10.3762/bjnano.15.53

**Published:** 2024-05-31

**Authors:** Priscila Jussiane Zambiazi, Dolores Ribeiro Ricci Lazar, Larissa Otubo, Rodrigo Fernando Brambilla de Souza, Almir Oliveira Neto, Cecilia Chaves Guedes-Silva

**Affiliations:** 1 Instituto de Pesquisas Energéticas e Nucleares, IPEN/CNEN-SP, Av. Prof. Lineu Prestes, 2242 Cidade Universitária, São Paulo, SP, CEP 05508-000, Brazil

**Keywords:** exfoliation, nanosheets, non-plasma method, titanium nitride

## Abstract

In this study, we present a novel approach for the exfoliation of titanium nitride (TiN) powders utilizing a rapid, facile, and environmentally friendly non-thermal plasma method. This method involves the use of an electric arc and nitrogen as the ambient gas at room temperature to generate ionized particles. These ionized species interact with the ceramic crystal of TiN, resulting in a pronounced structural expansion. The exfoliated TiN products were comprehensively characterized using transmission electron microscopy, X-ray diffraction, and Raman spectroscopy. Remarkably, the cubic crystal structure of TiN was effectively retained, while the (200) crystal plane d-spacing increased from 2.08 to 3.09 Å, accompanied by a reduction in crystallite size and alterations in Raman vibrational modes. Collectively, these findings provide compelling evidence for the successful exfoliation of TiN structures using our innovative non-thermal plasma method, opening up exciting possibilities for advanced material applications.

## Introduction

Since the groundbreaking discovery of graphene by Andre Geim and Konstantin Novoselov in 2004 [1], the field of nanostructures has witnessed remarkable advancements. Various methods to fabricate graphene, such as mechanical and chemical exfoliation, combined with innovative characterization techniques, have enabled the preparation of diverse layered two-dimensional (2D) materials with exceptional physical and chemical properties. These materials are typically exfoliated from three-dimensional (3D) layered crystals characterized by atomically thin layers held together by strong in-plane covalent bonds and weak out-of-plane van der Waals (vdW) forces. Apart from graphene, prominent 2D vdW materials include hexagonal boron nitride, transition metal dichalcogenides, talc nanoflakes, and black phosphorus [2–4].

However, because of the inherent chemical stability of 2D vdW materials under ambient conditions, researchers have also turned their attention to a distinct class of 2D materials. These novel materials consist of ultrathin nanosheets but are synthesized from non-van der Waals (n-vdW) bulk crystals featuring strong chemical bonds in all directions [5].

In general, n-vdW crystals combine the 2D structural characteristics with properties inherited from their parent crystals [6]. Their synthesis typically involves costly bottom-up processes. Some efforts have been successfully made to synthesize n-vdW 2D materials using liquid exfoliation techniques [7], albeit with relatively low yields. The first such material was obtained through electrostatically driven exfoliation of WO_3_ powder using bovine serum albumin as an exfoliating agent at pH 4 [8]. Subsequently, in 2018, Balan et al. [9] achieved the synthesis of hematene, a n-vdW 2D material, from natural iron ore hematite (α-Fe_2_O_3_) using liquid exfoliation. Unlike hematite, hematene exhibited ferromagnetic properties and demonstrated high visible-light photocatalytic activity when loaded onto titania nanotube arrays.

Titanium nitride (TiN) has gained recognition as an advanced engineering material because of its outstanding chemical and thermal stability, extreme hardness, and electrical conductivity [10–12]. On the nanoscale, TiN finds applications as additive in titanium alloys, as catalyst support material, as supercapacitor component, and as nanocoating for medical implants [13–16]. Furthermore, ultra-small TiN nanodots have been successfully obtained through liquid exfoliation methods, offering promising prospects for applications in photoacoustic imaging and photothermal therapy of tumors because of their satisfactory absorption characteristics in the second near-infrared (NIR-II) region [5]. The non-thermal plasma (NTP) synthesis method enables the fabrication of 2D materials by exfoliating massive materials. Hot electrons are shot into the crystal structure and cause repulsion between layers, resulting in few-layered 2D materials. Souza and coworkers [17] successfully demonstrated the production of few-layer hexagonal boron nitride nanosheets, starting from bulk boron nitride as the initial material. In addition to using fewer reagents compared to conventional methods involving acidic baths, oxidizers, ultrasound, multiple reaction steps, exfoliation, and washes, the presented method operates in a single step [18–19]. In this study, we explore the exfoliation of TiN powders using a rapid and promising technique involving a NTP generator, previously employed for synthesizing ultrathin BN nanosheets [17]. This method was selected for its potential to yield ultrathin nanosheets while preserving the original crystal structure of TiN.

## Experimental

**Figure 1:**
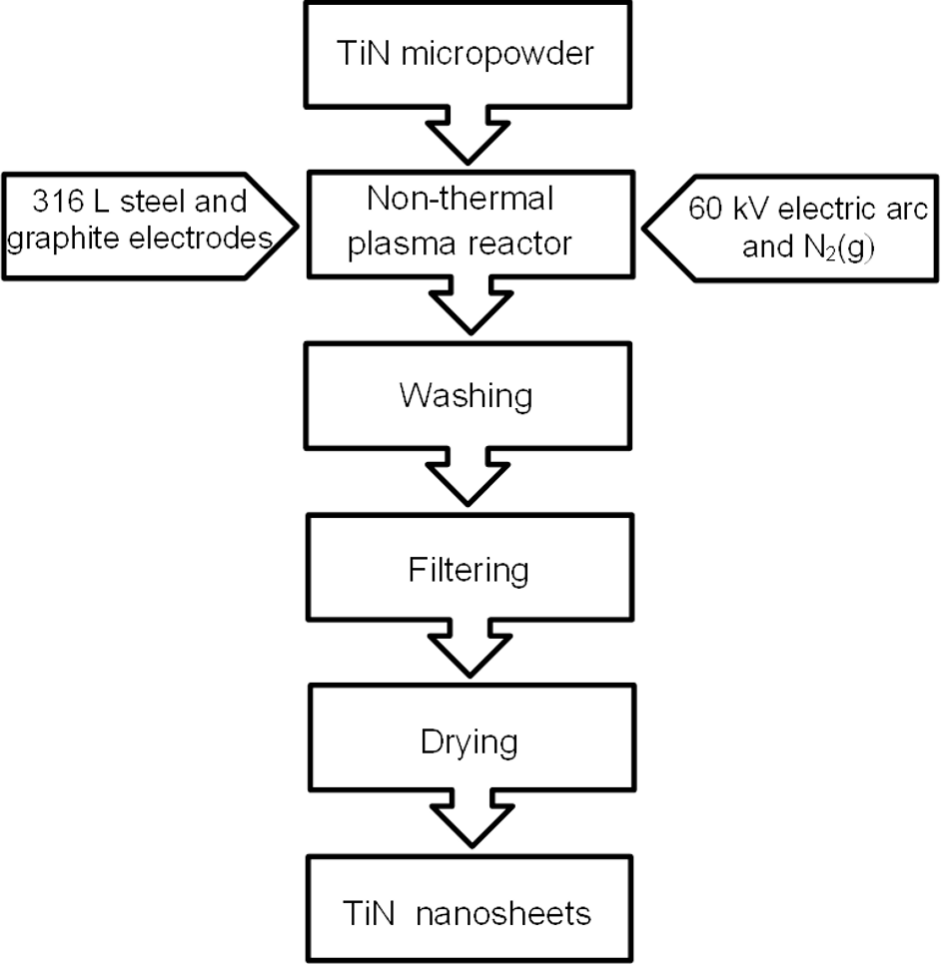
Flowchart of TiN nanosheet synthesis by the non-thermal plasma method.

The TiN nanosheets were synthesized utilizing a non-thermal plasma apparatus, as previously developed by de Souza et al. [17]. In this process, similarly as reported in [4], TiN powder with a particle size of approximately 1.0 μm and a purity of 98% (Alfa Aesar) was exposed to a 60 kV electric arc in the presence of a nitrogen gas flow. The arc was generated between a 316 L steel electrode and another electrode composed of graphite. The process lasted 60 min. Subsequently, the resultant material was collected, rinsed in a 1:1 mixture of water and isopropanol, and decanted for 24 h. Then, the liquid phase was filtered using a cellulose acetate membrane with 200 nm pores. The process was concluded by drying to obtain the TiN nanosheets. The flowchart of the synthesis procedure is shown in Figure 1.

The morphology of the exfoliated material was investigated by transmission electron microscopy (TEM) with a Jeol JEM-2100 electron microscope operating at 200 kV. X-ray diffraction (XRD) analysis was conducted using a diffractometer (Miniflex II) with Cu Kα radiation over a 2θ range of 20–90°, with a scan speed of 2° per minute. Furthermore, Raman spectra were obtained using a spectrometer (Horiba Scientific MacroRam Raman) equipped with a 785 nm laser source. These analytical techniques provided comprehensive insights into the structure and properties of the synthesized TiN nanosheets.

## Results and Discussion

**Figure 2:**
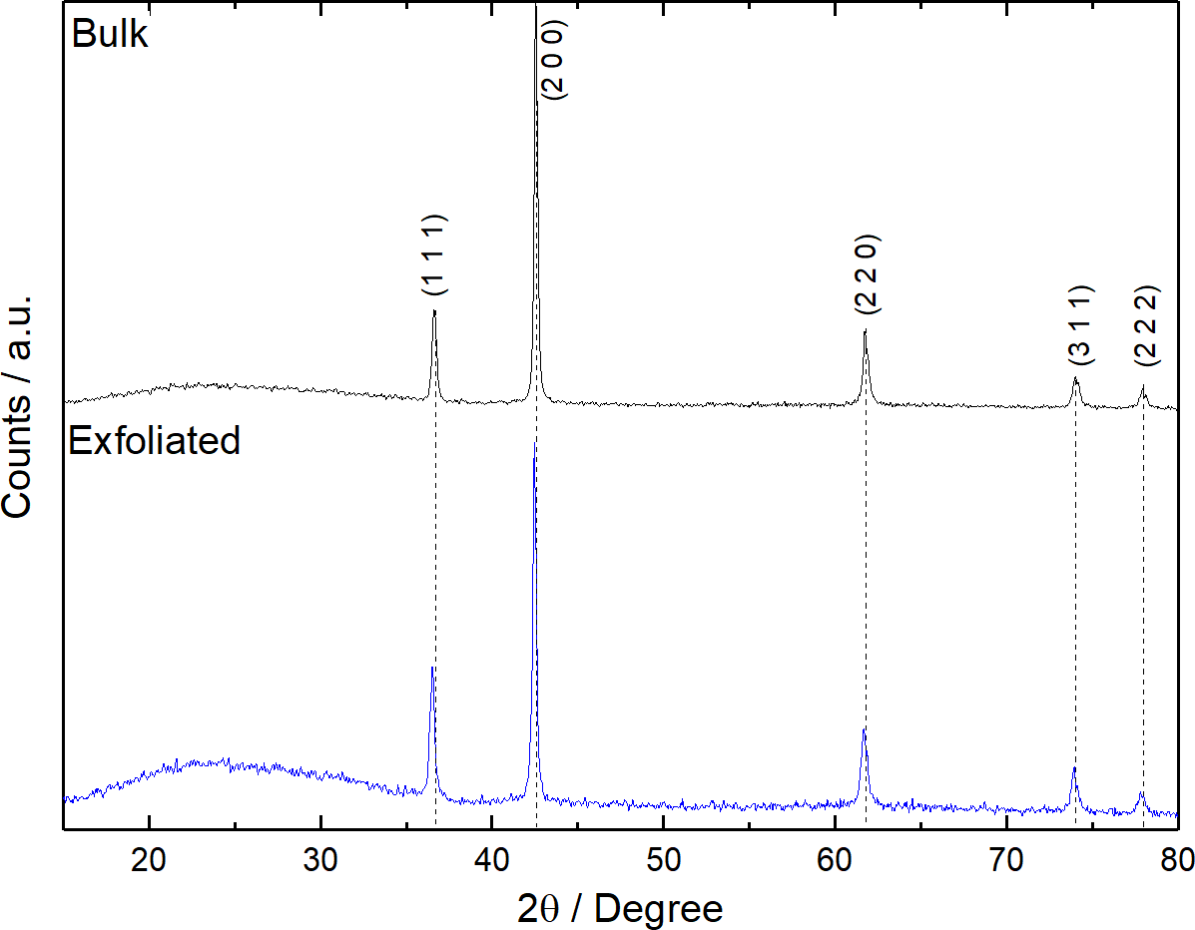
XRD patterns of the bulk and non-thermally processed powders.

Figure 2 shows the XRD patterns of both the bulk material and the TiN powder obtained from the non-thermal plasma process. The XRD pattern for cubic TiN, as compared to JCPDS # 87-0633, displays characteristic peaks at approximately 37°, 43°, 62°, 74°, and 78°. In contrast, the TiN processed by non-thermal plasma exhibited small shifts towards less positive values in the 2θ angles for the (111), (200), (220), (311), and (222) planes. This shift suggests an expansion of the lattice parameters of the crystal structure.

Additionally, the relative intensities of the (111), (200), (220), (311), and (222) planes were changed for TiN processed by non-thermal plasma. The planes showed relative intensities of 0.47, 1.00, 0.44, 0.22, and 0.13, respectively, while the corresponding values for bulk TiN were 0.32, 1.00, 0.39, 0.29, and 0.19. This discrepancy indicates that the non-thermal plasma reduced the abundance of the (200) plane while favoring the emergence of the (111) and (220) faces.

Changes in the relative plane intensities and lattice parameters strongly suggest that the non-thermal plasma method induced structural modifications in the TiN material. For the bulk material, FWHM (full width at half maximum) measurements were 0.28, 0.22, 0.31, 0.35, and 0.37 for the (111), (200), (220), (311), and (222) crystallographic planes, respectively. In contrast, the exfoliated material exhibited FWHM values of 0.30, 0.24, 0.44, 0.43, and 0.41 for the same crystallographic planes. These FWHM results suggest a reduction in the crystallite size for the exfoliated material.

Additionally, a slight but noteworthy shift in the peak positions, approximately 0.1° less positive, was observed in the X-ray diffractograms for the exfoliated material. This shift aligns with the indications of reduced crystallite size and is consistent with the outcomes reported by de Souza et al. [17], who obtained few layers of hexagonal BN using the same exfoliation method. These combined findings highlight the structural changes during the non-thermal plasma exfoliation process, further supporting the successful transformation of bulk TiN into nanosheets.

**Figure 3:**
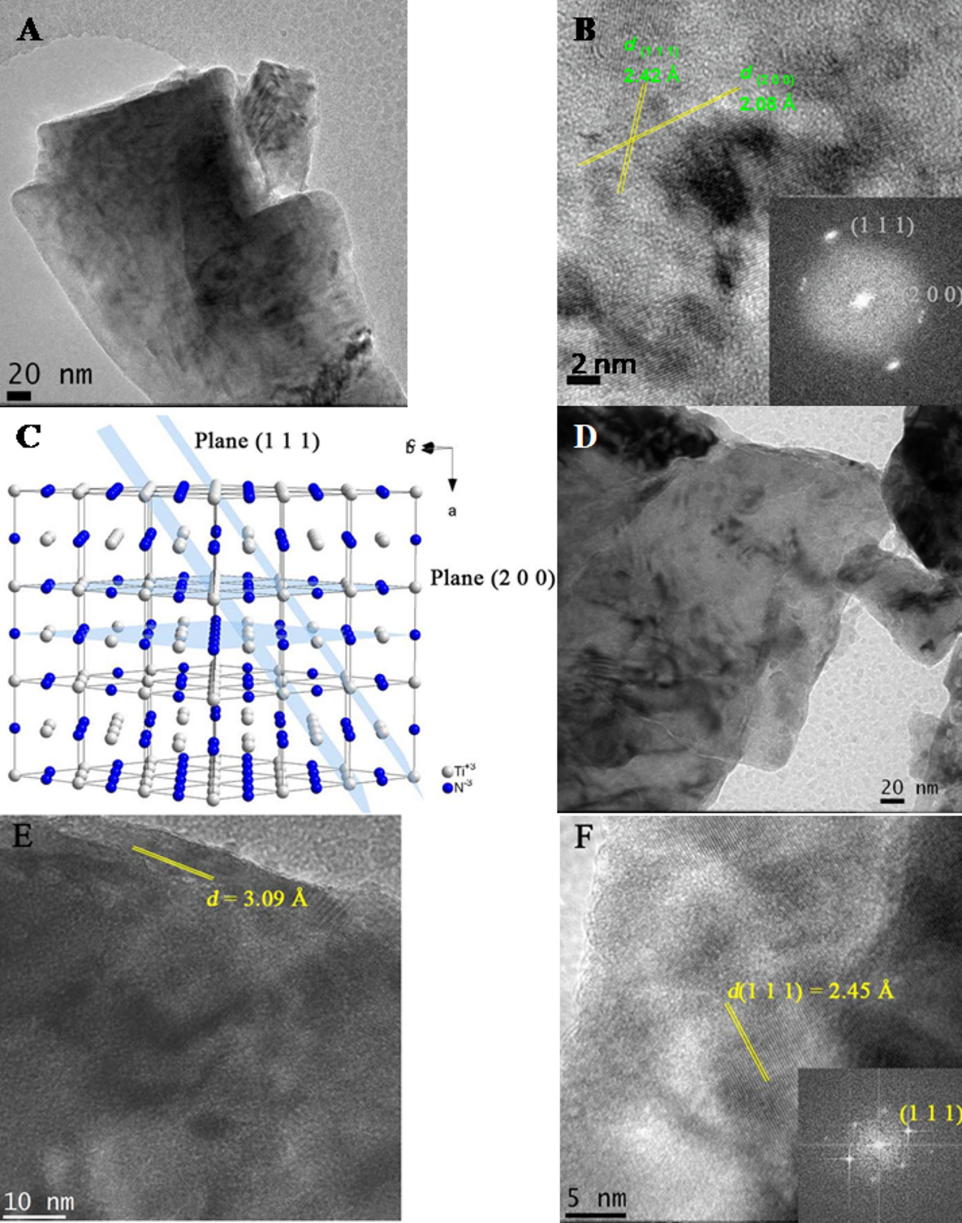
HRTEM images and crystal structure representation of TiN before exfoliation (A, B), representation of TiN planes (C), and HRTEM images and crystal structure after (D–F) exfoliation. Crystallographic planes and d-spacings are shown.

Figure 3 shows high-resolution transmission electron microscopy (HRTEM) images of TiN before and after non-thermal plasma exfoliation. In Figure 3a, 3D TiN blocks are visible. With higher magnification (Figure 3b), fringes with interplanar distances corresponding mainly to the (111) and (200) planes are observed. In Figure 3d, with the same magnification for the exfoliated material, a higher transparency to the microscope beam is observed, indicating fewer scattering centers. When comparing Figure 3a and Figure 3d, it is clear that the initial compact 3D TiN blocks underwent a transformation and evolved into plate-like thin layers.

The high-resolution image in Figure 3f shows a detailed view of the materials structure, revealing that it retained some crystallinity, typical of 2D nanofilms. This retention of crystalline structure was further confirmed by fast Fourier transform (FFT) analysis. Notably, the d-spacing of the (200) crystal plane increased from 2.08 to 3.09 Å (as observed in Figure 3b and Figure 3e), which is an indication of the successful exfoliation process. These observations are in line with the XRD and FWHM results.

Moreover, it is worth noting that the exfoliation of the TiN crystal structure occurred along the [111] crystallographic axis. This directional aspect is significant in understanding the structural changes during the plasma-induced non-thermal exfoliation of TiN.

**Figure 4:**
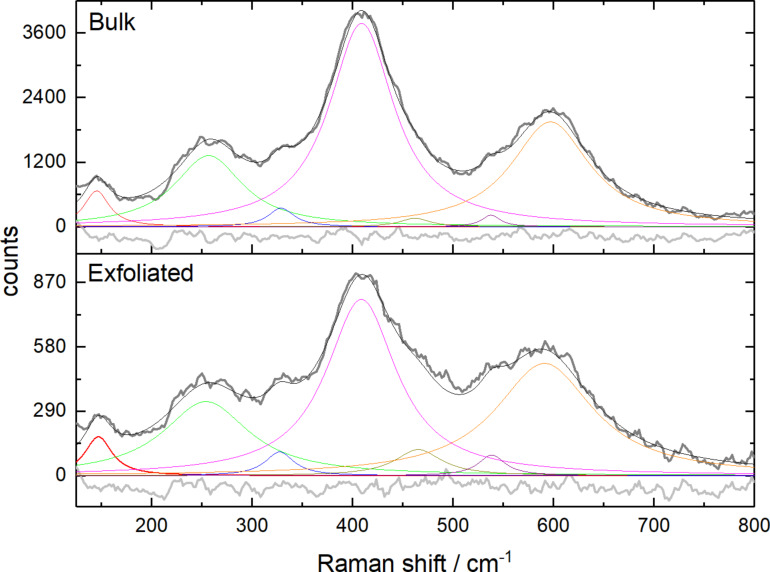
Raman spectra of the bulk and the non-thermally processed powders.

Figure 4 presents the Raman spectra of both bulk and exfoliated TiN samples. In these spectra, distinctive bands are observed at approximately 145, 253, 407, and 592 cm^−1^, corresponding to various vibrational modes. These include the first-order transverse acoustic (TA), longitudinal acoustic (LA), second-order acoustic (2A), and transverse optical (TO) modes, respectively [20–21].

The presence of a low-intensity peak around 145 cm^−1^ may be associated with the presence of rutile, possibly due to surface oxidation of the powder. This observation can explain the presence of the 340 cm^−1^ peak, indicative of anatase formation. Additionally, the Raman spectra reveal vibrations resulting from nitrogen and titanium deficiencies within the TiN structure. Specifically, the peak at 253 cm^−1^ is attributed to the vibration of heavy titanium ions around nitrogen vacancies, while the 592 cm^−1^ peak arises from nitrogen ion vibrations near titanium vacancies [22–24].

Remarkably, the Raman spectra for both bulk and exfoliated TiN samples are similar. This consistency suggests that the non-thermal plasma exfoliation process did not induce significant alterations in the structure and chemical composition of TiN. However, slight changes are evident for the exfoliated sample, mainly in shift and peak width of the center band. These observations are corroborated by the increment in the full width at half maximum (FWHM) of the Raman spectra and a decrease in peak intensity, all indicative of reduced crystallinity and the presence of internal stress (as detailed in Table 1) [25]. The Raman spectra are shown only up to 800 cm^−1^ because no other bands were observed at higher wavenumbers. This is an indication of no carbon contamination, which would be observed around 1300–1400 cm^−1^ and 1550–1600 cm^−1^ [26].

**Table 1:** Raman frequency and linewidth values of bulk and non-thermally processed powders.

Material	Vibration					
	TA		LA		TO	
	center band (cm^−1^)	FWHM (cm^−1^)	center band (cm^−1^)	FWHM (cm^−1^)	center band (cm^−1^)	FWHM (cm^−1^)

bulk	145	31	253	83	591	95
exfoliated	147	33	254	104	597	118

The relaxation in the vibrational modes, as evidenced by the Raman spectra, serves as a compelling indicator of the successful exfoliation. It is in good agreement with XRD and TEM results, collectively reinforcing that the non-thermal plasma method was effective in preserving the bulk chemical composition and structure, albeit with slight alterations in the crystallinity and the extent of internal stress.

## Conclusion

We have successfully produced titanium nitride nanosheets by a non-thermal plasma exfoliation process starting from micrometer-sized TiN powder. The resulting product exhibited a plate-like thin film morphology with an enlarged d-spacing of the (200) crystallographic planes and a relaxation of the Raman vibrational modes. While the cubic structure and chemical composition of TiN were preserved, the crystallite size was reduced. These TiN nanosheets have a great potential for a range of cutting-edge applications in fields such as medicine, catalysis, and energy-related technologies. Hence, further exploration and development of these TiN nanosheets are expected to yield significant advancements in materials science and technology.
